# PIEZO1 channels in cutaneous free nerve endings: novel insights into itch-scratch-mechanisms

**DOI:** 10.1038/s41392-022-01271-w

**Published:** 2023-01-02

**Authors:** Manuel Pedro Pereira, Henning Wiegmann, Sonja Ständer

**Affiliations:** Department of Dermatology and Center for Chronic Pruritus, University Hospital Münster, 48149 Münster, Germany

**Keywords:** Translational research, Neuroscience

A recent study published in *Nature* by Hill et al.^1^ identified mechano-sensitive PIEZO1 ion channels in pruriceptive neurons. Remarkably, PIEZO1 is involved in acute itch transmission and in mechanical alloknesis in chronic pruritus (CP) states.

Itch is defined as an unpleasant sensation leading to the desire to scratch. Scratching is a mechanical skin stimulation, which suppresses itch, however, scratching can also elicit itch itself as in atopic dermatitis (AD). In some entities, a severe itch-scratch-cycle develops, as for instance in chronic nodular prurigo (CNPG).^2^ More, strong mechanical stimuli may induce pruritic dermatoses in predisposed patients (for example inducible urticaria). Patients with CP often perceive light touch as itch; termed mechanical alloknesis. This occurs in non-lesional or in inflammatory skin such as eczemas. Accordingly, mechanically induced itch is a relevant observation in CP patients but the different types of mechanical itch are still poorly understood.

**Fig. 1:**
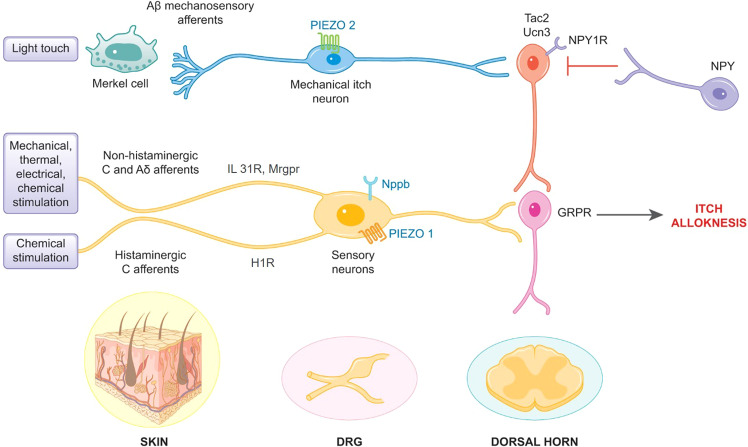
Presumed mechanical itch transduction pathways. Light tactile stimuli activate Aβ LTMR, which convey pruriceptive signals to spinal excitatory interneurons expressing Ucn3, Tac2, or NPY1R. Spinal inhibitory NPY expressing interneurons suppress this pathway under physiological conditions, but may contribute to itch and alloknesis in a pathological state. Inhibitory spinal interneurons are modulated by input from Merkel/PIEZO2 expressing Aβ LTMR. In CP states, this gate control is lost and light touch is perceived as itch (mechanical alloknesis). Mechanical stimulation can, however, also induce itch and alloknesis via Nppb-positive DRG neurons expressing mechano-sensitive PIEZO1 channels. These fibers also react to histaminergic and non-histaminergic chemical pruritogens. Conversely, in physiological conditions, gentle mechanical stimulation at a pleasurable intensity and pace (affective touch) are mediated by low-threshold mechano-sensitive C-fibers of hairy skin, projecting to lamina I-spinothalamic pathways. CP chronic pruritus, DRG dorsal root ganglion, GRPR gastrin-release peptide receptor, H1R histamine 1 receptor, IL 31R interleukin 31 receptor, LTMR low-threshold mechanoreceptors, Mrgpr MAS-related G-protein coupled receptors, Nppb natriuretic polypeptide precursor B, NPY neuropeptide Y, NPY1R neuropeptide Y1 receptor, Tac2 tachykinin 2, Ucn3 urocortin 3. This figure was edited by Elsevier Illustration Service

In the skin, itch can be induced by chemical, thermal, electrical and mechanical stimuli at free nerve endings. Pruritogens target histaminergic, slow-conducting C-fibers and non-histaminergic, mechano-sensitive C- and Aδ-fibers (Fig. 1). In recent years, the neuroimmune crosstalk involved in chemical itch induction by interleukins was well characterized fostering the development of novel therapies in inflammatory dermatoses.^2^ For example, interleukin 31 (IL 31) induces itch at sensory neurons and mediates inflammation; corresponding receptor blockade with an injectable antibody is effective in AD and CNPG.^2^ Itch signals are transmitted via free nerve endings and dorsal root ganglion (DRG) to the dorsal horn of the spinal cord. Among other proteins, the natriuretic polypeptide precursor B (Nppb) mediates the transmission between DRG and spinal interneurons. A chain of interneurons modulates and further transports the itch information to upper brain centers.

Interestingly, the spinal circuitry is involved in the conversion of the sensation of light touch into itch. Aβ-low-threshold mechanoreceptors (LTMR) activated by non-noxious mechanical stimulation transmit pruriceptive signals to excitatory interneurons at spinal level, which express urocortin 3 (Ucn3), tachykinin 2 (Tac2) or neuropeptide Y1 receptor (NPY1R). A subpopulation of inhibitory neuropeptide Y (NPY) interneurons suppress this pathway, however, under pathological conditions this inhibitory mechanism is lost. Spinal excitatory Tac2 and NPY1R interneurons connect with itch specific gastrin-release peptide receptor (GRPR) expressing neurons, which further transmit the itch signal to upper centers, arguing for an intersection of mechanical and chemical itch transduction pathways at central level. In CP states, neuronal sensitization occurs via augmented activity of excitatory Ucn3 neurons and reduced activity of inhibitory NPY interneurons.

In the skin, different aspects of tactile and mechanical sensations exist (light touch, emotional touch, perception of pressure, vibration, slip, texture) under physiological conditions, which are mediated by LTMR.^3^ For example, Aβ nerve endings are associated with Merkel cells (MC) for mechanoreception in the skin and mediate discriminative touch under physiological conditions. More precisely, MC at the basal level of the epidermis are innervated by Aβ-LTMRs expressing PIEZO2 ion channels. The PIEZO protein family are mechano-sensitive calcium channels first described in 2010.^4^ The MC/PIEZO2 channel unit modulates mechanical alloknesis under pathological conditions, as the loss of MC or the genetic ablation of PIEZO2 channels is associated with enhanced alloknesis in a murine dry skin model.^5^ Hence, in physiological states, tactile skin stimulation is perceived as light touch due to modulation of inhibitory spinal interneurons by input from MC/PIEZO2 expressing Aβ-LTMRs. This gate control homeostatic mechanism is lost under pathological CP conditions, for instance due to degeneration of MC, resulting in mechanical alloknesis. This phenomenon is oftentimes observed in CP patients, as light touch (e.g., due to rubbing of clothes on skin) may induce an intolerable itch.

However, this knowledge on the role of PIEZO2 does not explain the different types of mechanical itch as, for example, in mechanical-induced urticaria where histaminergic C-fibers are involved. Hill and colleagues^1^ identified the exciting role of PIEZO1 in mechanical itch mediated by sensory neurons. They identified PIEZO1 ion channels in murine and human Nppb-positive DRG neurons and murine MAS-related G-protein coupled receptor (Mrgpr) A3-positive nerves. Mrgpr receptors are highly associated with itch transmission. PIEZO1 channels were shown to be functionally active in pruriceptive neurons, as PIEZO1 channels responded to stimulation with histamine and β-alanine (Mrgpr-agonist).^1^ Murine PIEZO1 knockout (KO; Piezo1fl/fl;PirtCre+/− and Piezo1fl/fl;SstCre+/−) experiments confirmed the role of PIEZO1 in alloknesis (scratching induced with stimulation with von Frey filaments) using the pruritogen histamine and IL 31 to create a CP state. This argues for the fact that PIEZO1 is present on histaminergic and non-histaminergic nerve fibers. More, the authors showed that PIEZO1 can trigger acute itch as injection of Yoda1 (PIEZO1 agonist) leads to scratching in animals. In addition, in KO mice, the extent of itch induction by histamine and IL 31 was reduced. However, experimentally induced skin inflammation (mimicking AD) did not alter itch sensation in KO mice suggesting that PIEZO1 channels are not always required in chemical itch generation. Finally, blocking of PIEZO1 channels after intraperitoneal injection of the toxin GsMTx4 reduced mechanically evoked scratching in naive mice.

Based on these experiments, the authors conclude that subsets of peripheral slow conduction C-fibers expressing PIEZO1 promote itch and alloknesis upon mechanical stimulation. These nerve fibers are also sensitive to histaminergic and non-histaminergic chemical pruritogens, contrary to the previously described PIEZO2 channels, which are solely responsive to mechanical stimuli. The exciting novel breakthrough by Hill and colleagues enhances our understanding of peripheral mechanical itch transduction mechanisms especially in CP states. PIEZO1 is clearly linked to alloknesis mediated via free nerve endings. Regarding the physiologic significance of this finding, it can be speculated that the channel serves to augment acute itch signals and thereby scratching behavior along with infections or allergies of the skin. The aim might be to enforce removal of the skin damaging substances or to guide consciousness to an injured skin area. In the state of CP, PIEZO1 mediated alloknesis perpetuates itch duration and leads to higher itch intensity. The clinical relevance of this important paper is thus clear; target validation in humans including determination of the clinical efficacy of PIEZO1 blockade will be a task of the coming years.
